# Association Between Urinary Bisphenols and Body Composition Among American Adults: Cross-Sectional National Health and Nutrition Examination Survey Study

**DOI:** 10.2196/49652

**Published:** 2023-09-19

**Authors:** Jiakun Li, Zilong Zhang, Chichen Zhang, Qiyu Zhu, Jing Zhao, Hui Zong, Qi Deng, Jiaming Zheng, Erman Wu, Rongrong Wu, Tong Tang, Yihang Zhang, Qiaosen Dong, Yifan Li, Jiao Wang, Lu Yang, Shi Qiu, Bairong Shen, Qiang Wei

**Affiliations:** 1 Department of Urology West China Hospital Sichuan University Chengdu China; 2 Institutes for Systems Genetics Frontiers Science Center for Disease-related Molecular Network West China Hospital, Sichuan University Chengdu China

**Keywords:** bisphenols, body composition, environmental pollutants, public health, medical informatics

## Abstract

**Background:**

Bisphenol A (BPA), bisphenol S (BPS), and bisphenol F (BPF) are widely used in various consumer products. They are environmental contaminants with estrogenic properties that have been linked to various health outcomes. Understanding their impact on body composition is crucial for identifying potential health risks and developing preventive strategies. However, most current studies have only focused on their relationship with BMI.

**Objective:**

This study aimed to investigate the association between urinary levels of BPA, BPS, and BPF and body composition, including BMI, lean mass, and fat mass, in a large population-based sample.

**Methods:**

We conducted a cross-sectional analysis using data from the National Health and Nutrition Examination Survey 2003-2016. Body composition data were assessed using dual-energy X-ray absorptiometry, which provided precise measurements of lean mass, fat mass, and other indicators. We used multivariate linear regression models to estimate the associations, adjusting for potential confounders such as age, gender, race, socioeconomic factors, and lifestyle variables.

**Results:**

The results revealed significant associations between bisphenol exposure and body composition. After adjusting for covariates, BPS showed a positive association with BMI, with quartiles 3 and 4 having 0.91 (95% CI 0.34-1.48) and 1.15 (95% CI 0.55-1.74) higher BMI, respectively, compared with quartile 1 (*P*<.001). BPA was negatively associated with total lean mass (TLM) and appendicular lean mass, with quartiles 2, 3, and 4 having –7.85 (95% CI –11.44 to –4.25), –12.33 (95% CI –16.12 to –8.54), and –11.08 (95% CI –15.16 to –7.01) lower TLM, respectively, compared with quartile 1 (*P*<.001). BPS was negatively associated with TLM, with quartiles 3 (β=–10.53, 95% CI –16.98 to –4.08) and 4 (β=–11.14, 95% CI –17.83 to –4.45) having significantly lower TLM (*P*=.005). Both BPA and BPS showed a positive dose-response relationship with trunk fat (BPA: *P*=.002; BPS: *P*<.001) and total fat (BPA: *P*<.001; BPS: *P*=.01). No significant association was found between BPF and any body composition parameter.

**Conclusions:**

This large-sample study highlights the associations between urinary levels of BPA and BPS and alterations in body composition, including changes in lean mass, fat mass, and regional fat distribution. These findings underscore the importance of understanding the potential health risks associated with bisphenol exposure and emphasize the need for targeted interventions to mitigate adverse effects on body composition.

## Introduction

### Background

Bisphenol A (BPA) is an endocrine disrupter used globally in the manufacture of various plastic products including food containers, baby bottles, toys, and medical supplies [1,2]. It not only exhibits estrogen-like effects but also antagonizes thyroid hormone receptors, which may lead to adverse metabolic effects and diseases such as cardiovascular conditions, tumors, and other metabolism-related ailments [2,3].

Countries including Canada, the European Union, the United States, and China have prohibited the use of BPA in certain plastic products due to these health risks. The US Food and Drug Administration has set strict acceptable daily intake levels for BPA, but studies have indicated that even levels below the acceptable daily intake can contribute to obesity and related metabolic disturbances. Consequently, BPA substitutes, bisphenol S (BPS) and bisphenol F (BPF), were introduced [4].

These BPA derivatives (BPS and BPF), obtained by replacing the propane group in the molecular structure of BPA, are found to have estrogenic effects similar to those of BPA, indicating that they could also be unsafe [4]. They are prevalent in everyday packaging products and can be ingested through diet, air inhalation, or skin contact [5]. These chemicals can interfere with the nuclear hormone receptor signaling pathway, thereby affecting adipocyte proliferation and differentiation, which could potentially lead to a variety of metabolic diseases [4].

Body composition, including not only BMI but also fat mass and lean body mass, is a critical aspect of health [6]. Excessive body fat has been linked to adverse health outcomes including breast cancer [7,8] and cardiovascular risk factors [9]. Abdominal fat distribution is associated with an increased risk of type 2 diabetes [10], arterial stiffness [11], and cardiovascular disease [12,13], whereas lean body mass, predominantly composed of skeletal muscles, influences bone health and optimal aging [14,15]. Skeletal muscle is the largest and most plastic component of lean body mass (accounting for approximately 50% of lean body mass). Therefore, changes in lean body mass are primarily attributed to skeletal muscle mass. The maintenance of skeletal muscle is the basis of exercise, energy balance, and overall quality of life [16,17] and is critical for the risk of heart metabolic diseases [18].

Studies have shown that BPA promotes adipocyte differentiation and insulin resistance [19,20]. Previous studies have explored the relationship between urinary BPA and body composition, but these studies have been largely limited to specific populations, such as women who are nonobese and in the premenopausal stage or older adults [21,22]. At present, research on the association between BPA analogs (BPS and BPF) and body composition is scarce.

### Objective

We aimed to bridge this gap by assessing the relationship between urinary BPA, BPS, BPF, and body composition in adult National Health and Nutrition Examination Survey (NHANES) participants using dual-energy X-ray absorptiometry (DXA) scans. By doing so, we hope to provide valuable insights into the health implications of BPA and its analogs in a broader population.

## Methods

### Patients Selection

The NHANES database is a research program designed to assess the health and nutritional status of nonhospitalized adults and children in the United States. The unique feature of this survey is that it combines interviews and physical examination [23]. More specifically, nearly 7000 residents are randomly selected and invited to the NHANES interviews every year. The interviews include population-, socioeconomic status–, diet-, and health-related issues. The examination section includes medical, dental, and physiological measurements as well as laboratory tests conducted by trained medical personnel.

Because NHANES used the DXA method to measure body composition, only people aged 8 to 59 years were measured in this database. In addition, pregnant women, people who have used radiocontrast agent (barium) in the past 7 days, and people who weigh >204 kg or are taller than 195.58 cm have also been initially excluded from the NHANES surveys. Our study included and analyzed data from 7 NHANES survey cycles (2003-2004, 2005-2006, 2007-2008, 2009-2010, 2011-2012, 2013-2014, and 2015-2016). Missing data on bisphenols (BPA, BPS, and BPF); BMI; or body compositions (total lean mass [TLM], appendicular lean mass [ALM], trunk fat [TRF], bone mineral content [BMC], and total fat [TOF]) were excluded.

Exclusion criteria were as follows: (1) data regarding BPA, BPS, or BPA were missing; (2) body composition data were missing; (3) the percentage of bisphenols (BPA, BPS, or BPF) >95% was defined as an outlier, and outliers were excluded.

**Figure 1:**
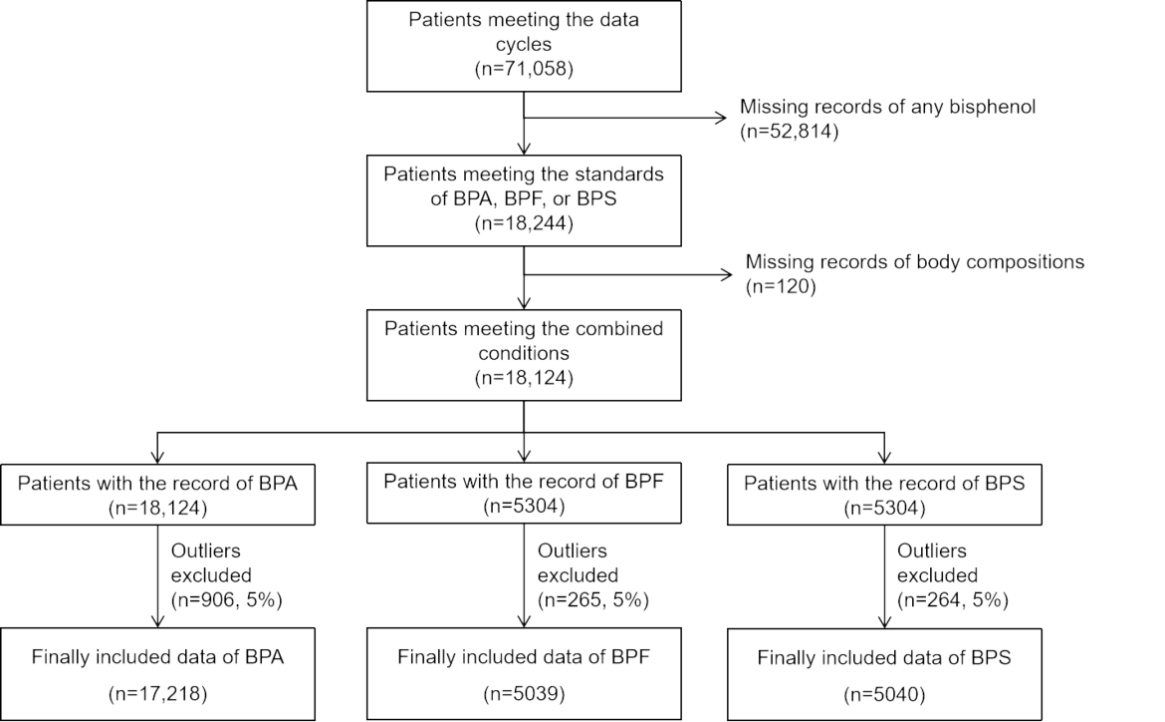
Flowchart of inclusion and exclusion criteria among American adults from the National Health and Nutrition Examination Survey 2003 to 2016. BPA: bisphenol A; BPF: bisphenol F; BPS: bisphenol S.

We included 71,058 original records at first. After excluding the missing data and outliers, 17,218 data were finally included in the BPA analysis study, whereas 5039 and 5040 data were finally included in the BPF and BPS analysis studies, respectively. Detailed inclusion and exclusion criteria are shown in Figure 1.

### Ethical Considerations

All study participants signed written informed consent forms, and the NHANES data collection was approved by the Research Ethics Review Board of the National Center for Health Statistics [24]. The NHANES has stringent consent protocols and procedures for ensuring confidentiality and protection against identification. This study was a secondary data analysis, which lacked personal identifiers, and the NHANES data are free for public use and available on the web [23]. Therefore, this study did not require an institutional review board review [24].

### Measurement of Bisphenols

A sensitive phenol-detection method was used in this study [25]. This method is a web-based solid-phase extraction method coupled with high-performance liquid chromatography and tandem mass spectrometry. In addition, urine creatinine in each urine sample was measured and used to adjust the urine dilution. The specifics of the bisphenol measurements were provided by NHANES [26]. We sought to access the NHANES, gleaned the relevant data successfully, and recorded them in their database duly, corresponding to the measurements undertaken.

### Measurement of Body Composition

The body composition included TLM, ALM, TRF, BMC, and TOF. DXA is the most widely accepted method for measuring body composition, partly because of its speed, ease of use, and low radiation exposure [27,28]. The NHANES DXA examination provides data on body composition (bone and soft tissue) as well as data on age, gender, and race and ethnic groups, which are generally representative of the United States, to study the association between body composition and other health conditions and risk factors, such as cardiovascular disease, diabetes, hypertension, and patterns of physical activity and dietary intake. DXA scans provide bone and soft tissue measurements of the entire body, arms and legs, trunk, and head.

The whole-body scan was obtained using the Hologic Discovery model A densitometer (version Apex 3.2; Hologic) [29]. The radiation exposure of whole-body DXA scanning was extremely low, less than 20 µSv. DXA examinations were performed by trained and certified radiologists. Further details of the DXA examination protocol are recorded in the Body Composition Procedure Manual on the NHANES website.

### Covariates

Age, race, education level, marital status, and income:poverty ratio were among the demographic factors recorded. The income:poverty ratio was determined by dividing household income by the poverty threshold, specifically the size of the household, in the corresponding year and the state. BMI, which was determined by dividing weight by height squared, was one of the variables examined. Laboratory test results included serum calcium and phosphorus levels. The variables in the questionnaire included the coronary artery disease (CAD) score, alcohol use, physical activity, and smoking status. The CAD score was obtained from the Cardiovascular Disease and Health section of the NHANES database defined by the Rose questionnaire [30]. Energy (kcal) was calculated as the energy intake in food and beverages consumed within 24 hours before the interview (midnight to midnight), based on the energy of each food item in the US Department of Agriculture Food and Nutrient Database for Dietary Studies.

### Data Analyses

We used appropriate survey procedures to explain the complex sampling design and weights of the NHANES [31]. The weights of the NHANES data consider several survey features, including the differential probabilities of selection for the sampling domains, survey nonresponse, and differences between the final sample distribution and the target population distribution. Each of the 3 levels of data collection for the NHANES (screening, interview, and examination) had a response rate. Subsequently, the sample weights were calculated for each level of data collection by the statistician of the National Center for Health Statistics. The selection of weights was carried out by the researchers based on the NHANES analysis protocol and the study content. This study selected the full sample of the 2-year mobile examination center exam weight. When we combined the weights of 7 cycles of the NHANES study, we divided the raw weight data by 7. Continuous variables were expressed as mean (95% CI), whereas categorical variables were expressed as percentages. The Student 2-tailed *t* test or Mann-Whitney *U* test was used to compare continuous variables, and the chi-square test or Fisher exact test was used to compare categorical variables.

A multivariate linear regression model was used to estimate the association between the body composition of phenolic substances, including an unadjusted model (nonadjusted); a minimally adjusted model (adjust I; adjusted only for gender, age, race, energy [kcal], and creatinine, urine [mg/100 mL]); and a fully adjusted model (adjust II; adjusted for gender, age, race, ratio of family income to poverty, education level, marital status, CAD score, smoking status, alcohol intake, physical activity, energy [kcal], and creatinine, urine (mg/100 mL]).

In the aforementioned models, body composition and body weight were used for adjustment in all analyses, except BMI and body weight. In addition, for missing covariate data, the method of missing value interpolation was supplemented. The interpolation threshold was set to 5%. Phenolic substances (BPA, BPS, or BPF) were considered continuous independent variables and categorical variables (divided into quartiles), and the lowest quartile was used as the reference. As the detection rate of BPF was low, quartiles 1 and 2 of the original data were combined to form a new quartile.

We performed tests for linear trends by entering the median value of each category as a continuous variable in the models. Interactive and stratified analyses were performed. Each stratification was adjusted for all factors (gender, age, alcohol intake, race, smoking status, and physical activity) except for the stratification factor itself.

All statistical analyses were performed using the software packages R (version 4.2.0; The R Foundation) and Empower (X&Y Solutions Inc) [32]. Double-tailed *P*<.05 was considered statistically significant.

## Results

In total, 18,124 participants were selected for our study. Among them, 8988 were male and 9133 were female, the median age was 39.49 (IQR 38.94-40.04) years, and the median BMI was 27.22 (IQR 27.03-27.41) kg/m^2^. Among the 18,124 participants, we conducted statistical analysis on 17,218 (95%) participants with BPA data, 5039 (27.8%) people with BPS data, and 5040 (27.8%) people with BPF data according to the information from the NHANES database. In addition, urinary BPA, BPS, and BPF were 2.27 (95% CI, 2.22-2.33), 0.70 (95% CI, 0.65-0.75), and 0.72 (95% CI, 0.66-0.78) ng/mL, respectively. Detailed characteristics of the participants are presented in Table 1.

**Table 1:** Characteristics, including baseline, urinary bisphenols and body compositions, of American adults from the National Health and Nutrition Examination Survey 2003-2016.

Characteristics^a^		Total (n=18,124)	BPA^b^ (n=17,218)	BPS^c^ (n=5039)	BPF^d^ (n=5040)
Age (years), median (IQR)		39.49 (38.94-40.04)	39.65 (39.10-40.21)	40.13 (38.99-41.27)	40.18 (39.02-41.35)
Energy (kcal), mean (95% CI)		2171.8 (2149.81-2193.79)	2172 (2150.07-2193.94)	2120.14 (2078.95-2161.34)	2110.17 (2067.34-2152.99)
Creatinine, urine (mg/100 mL), mean (95% CI)		123.65 (121.61-125.69)	120.65 (118.55-122.76)	122.32 (118.30-126.34)	122.1 (117.95-126.24)
Weight (kg), mean (95% CI)		75.67 (75.09-76.25)	75.58 (74.99-76.17)	76.23 (74.99-77.46)	75.92 (74.73-77.11)
BMI (kg/m^2^), mean (95% CI)		27.22 (27.03-27.41)	27.18 (26.99-27.38)	27.64 (27.20-28.07)	27.61 (27.19-28.03)
TLM^e^ (g), mean (95% CI)		48,965.91 (48,524.82-49,406.99)	48,954.92 (48,507.70-49,402.14)	49,114.27 (48,203.47-50,025.07)	48,934.37 (48,007.14-49,861.59)
ALM^f^ (g), mean (95% CI)		21,163.06 (20,942.60-21,383.52)	21,148.1 (20,926.20-21,370.00)	20,985.1 (20,546.07-21,424.14)	20,932.29 (20,486.99-21,377.58)
TRF^g^ (g), mean (95% CI)		12,231.43 (11,976.08-12,486.77)	12,176.15 (11,920.52-12,431.78)	12,095.54 (11,660.14-12,530.94)	12,072.51 (11,652.44-12,492.58)
BMC^h^ (g), mean (95% CI)		2260.29 (2242.46-2278.12)	2260.52 (2242.28-2278.76)	2191.89 (2164.24-2219.53)	2185.9 (2156.96-2214.85)
TOF^i^ (g), mean (95% CI)		25,522.09 (25,072.09-25,972.09)	25,442.36 (25,001.33-25,883.38)	25,447.67 (24,684.67-26,210.67)	25,319.4 (24,577.47-26,061.33)
Urinary bisphenols (ng/mL), mean (95% CI)		N/A^j^	2.27 (2.22-2.33)	0.7 (0.65-0.75)	0.72 (0.66-0.78)
**Gender, %**					
	Male	49.10	48.91	48.08	47.54
	Female	50.90	51.09	51.92	52.46
**Race, %**					
	Mexican American	9.40	9.48	10.18	10.65
	Other Hispanic	5.46	5.41	6.34	6.56
	Non-Hispanic Black	12.12	11.93	12.16	12.33
	Non-Hispanic White	65.68	65.81	62.21	61.04
	Other race	7.35	7.37	9.11	9.42
**Ratio of family income to poverty, %**					
	<1.3	23.49	23.13	24.51	25.54
	1.3-3.5	35.73	35.45	35.28	35
	>3.5	40.79	41.42	40.21	39.47
**Education level,** **%**					
	Less than high school	18.15	18.12	16.36	16.41
	High school or general educational development	22.20	21.89	20.09	20.45
	Above high school	59.65	59.99	63.55	63.13
**Marital status,** **%**					
	Married or living with partner	69.05	69.30	69.35	68.99
	Living alone	30.95	30.70	30.65	31.01
**CAD^k^ score, %**					
	0	62.07	62.08	59.20	60.07
	1	24.15	24.18	24.92	24.12
	2	13.79	13.74	15.88	15.80
**Smoking,** **%**					
	Never	54.54	54.76	57.76	57.78
	Former	28.03	27.96	27.98	27.73
	Current	17.43	17.29	14.25	14.48
**Alcohol intake (per day), %**					
	None	77	76.86	78.26	78.17
	Moderate	7.20	7.19	6.27	6.05
	Heavy	15.80	15.95	15.47	15.79
**Physical activity, %**					
	Less than moderate	39.11	38.91	42.73	42.96
	Moderate	15.69	15.66	10.19	10.46
	Vigorous	45.20	45.43	47.09	46.59

^a^The data showed for continuous variables were survey-weighted mean (95% CI) and for categorical variables, survey-weighted percentages (the absolute values cannot be provided due to being adjusted by weighting).

^b^BPA: bisphenol A.

^c^BPS: bisphenol S.

^d^BPF: bisphenol F.

^e^TLM: total lean mass.

^f^ALM: appendicular lean mass.

^g^TRF: trunk fat.

^h^BMC: bone mineral content.

^i^TOF: total fat.

^j^N/A: not applicable.

^k^CAD: coronary artery disease.

Associations between bisphenols (BPA, BPS, and BPF) and body compositions are presented in Table 2. A dose-response relationship was observed between BPA and BMI in the crude model (*P*<.001). The relationship did not exist after full adjustment; however, there was a positive association between BPS and BMI, with 0.91 and 1.15 higher BMI in quartiles 3 and 4, respectively, compared with quartile 1 (*P*<.001). In addition, there was also a dose-response relationship between BPA and TLM in the minimally and fully adjusted models (all *P*<.001), whereas a negative association was found between BPS and TLM after adjusting for all potential confounders, compared with quartile 1; participants in quartiles 3 and 4 had significantly lower TLM (quartile 3, model 2: β=–10.53, 95% CI –16.98 to –4.08; quartile 4, model 2: β=–11.14, 95% CI –17.83 to –4.45; *P*=.005). After adjusting for all confounders, a dose-response relationship was also found between BPA and ALM, with those in quartiles 2, 3, and 4 having significantly lower ALM than those in quartile 1 (quartile 2: β=–3.31, 95% CI –5.73 to –.90; quartile 3: β=–5.46, 95% CI –8.01 to –2.90; quartile 4: β=–4.80, 95% CI –7.55 to –2.05; *P*=.01). However, there was no substantial association between BPS and ALM. In addition, there was a dose-response relationship between BPA and TRF or TOF. For TRF, participants in quartiles 2, 3, and 4 (model 2: β=3.55, 95% CI 1.17-5.93; β=8.29, 95% CI 5.78-10.79; β=5.68, 95% CI 2.98-8.37, respectively; *P*=.002) had a significantly higher TRF than those at quartile 1. Similarly, there was a dose-response relationship between BPA and TOF (model 2: *P*<.001). For BPS, a positive association was identified between BPS and TRF or TOF; those in quartiles 3 and 4 had 5.16 and 8.41 higher TRF, 9.92 and 10.34 higher TOF when compared with quartile 1, respectively (for TRF, model 2: *P*<.001; for TOF, model 2: *P*=.01). Furthermore, although there was no significant association between BPA and BMC, there was a negative association between BPS and TOF (model 2: *P*<.001). After complete adjustment, there was no substantially association between BPF and body composition indicators (Table 2 and Multimedia Appendix 1).

**Table 2:** Association between bisphenols and body composition among American adults from the National Health and Nutrition Examination Survey 2003-2016.

				Nonadjusted				Adjusted model I^a^					Adjusted model II^b^		
				β (95% CI)		*P* value		β (95% CI)		*P* value		β (95% CI)			*P* value
**Urinary bisphenol A**															
	**BMI (kg/m^2^)**					<.001^c^				.23^c^					.11^c^
		0.14-0.7	Ref^d^				Ref				Ref				
		0.8-1.5	.62 (.31 to .92)		<.001		.31 (.02 to .59)		.04		.19 (–.09 to .46)			.19	
		1.6-3.1	1.19 (.89 to 1.49)		<.001		.46 (.16 to .76)		.002		.35 (.06 to .64)			.02	
		3.2-11	1.01 (.70 to 1.31)		<.001		–.01 (–.34 to .32)		.95		–.11 (–.43 to .20)			.48	
	**TLM^e^**					.11^c^				<.001^c^					<.001^c^
		0.14-0.7	Ref				Ref				Ref				
		0.8-1.5	–1.65 (–6.58 to 3.28)		.51		–8.96 (–12.66 to –5.26)		<.001		–7.85 (–11.44 to –4.25)			<.001	
		1.6-3.1	–2.15 (–7.05 to 2.76)		.39		–13.17 (–17.06 to –9.28)		<.001		–12.33 (–16.12 to –8.54)			<.001	
		3.2-11	3.00 (–1.98 to 7.97)		.24		–11.62 (–15.79 to –7.45)		<.001		–11.08 (–15.16 to –7.01)			<.001	
	**ALM^f^**					<.001^c^				.005^c^					.01^c^
		0.14-0.7	Ref				Ref				Ref				
		0.8-1.5	1.47 (–1.63 to 4.58)		.35		–4.07 (–6.53 to –1.61)		.001		–3.31 (–5.73 to –.90)			.007	
		1.6-3.1	2.93 (–.17 to 6.02)		.06		–5.85 (–8.44 to –3.25)		<.001		–5.46 (–8.01 to –2.90)			<.001	
		3.2-11	6.33 (3.19 to 9.47)		<.001		–5.43 (–8.22 to –2.65)		<.001		–4.80 (–7.55 to –2.05)			<.001	
	**TRF^g^**					.85^c^				<.001^c^					.002^c^
		0.14-0.7	Ref				Ref				Ref				
		0.8-1.5	1.63 (–1.37 to 4.63)		.29		4.53 (2.06 to 7.00)		<.001		3.55 (1.17 to 5.93)			.004	
		1.6-3.1	5.78 (2.79 to 8.76)		<.001		9.03 (6.44 to 11.63)		<.001		8.29 (5.78 to 10.79)			<.001	
		3.2-11	.76 (–2.27 to 3.79)		.62		6.66 (3.88 to 9.45)		<.001		5.68 (2.98 to 8.37)			<.001	
	**TOF^h^**					.23^c^				<.001^c^					<.001^c^
		0.14-0.7	Ref				Ref				Ref				
		0.8-1.5	2.13 (–2.99 to 7.25)		.42		9.66 (5.76 to 13.57)		<.001		8.58 (4.78 to 12.39)			<.001	
		1.6-3.1	3.73 (–1.37 to 8.83)		.15		15.07 (10.96 to 19.18)		<.001		14.19 (10.18 to 18.20)			<.001	
		3.2-11	–2.04 (–7.21 to 3.12)		.44		13.08 (8.68 to 17.48)		<.001		12.51 (8.20 to 16.82)			<.001	
**Urinary bisphenol S**															
	**BMI (kg/m^2^)**					<.001^c^				<.001^c^					<.001^c^
		0.07-0.1	Ref				Ref				Ref				
		0.2-0.3	.73 (.13 to 1.34)		.02		.27 (–.30 to .84)		.35		.26 (–.29 to .81)			.35	
		0.4-0.8	2.02 (1.42 to 2.62)		<.001		1.04 (.45 to 1.62)		<.001		.91 (.34 to 1.48)			.002	
		0.9-4.7	2.70 (2.10 to 3.31)		<.001		1.31 (.70 to 1.91)		<.001		1.15 (.55 to 1.74)			<.001	
	**TLM**					.006^c^				<.001^c^					.005^c^
		0.07-0.1	Ref				Ref				Ref				
		0.2-0.3	–2.53 (–10.95 to 5.88)		.56		–4.46 (–10.82 to 1.89)		.17		–3.81 (–10.05 to 2.44)			.23	
		0.4-0.8	–6.42 (–14.64 to 1.80)		.13		–12.79 (–19.32 to –6.25)		<.001		–10.53 (–16.98 to –4.08)			.001	
		0.9-4.7	–11.26 (–19.59 to –2.92)		.008		–13.51 (–20.28 to –6.74)		<.001		–11.14 (–17.83 to –4.45)			.001	
	**TRF**					<.001^c^				<.001^c^					<.001^c^
		0.07-0.1	Ref				Ref				Ref				
		0.2-0.3	1.92 (–3.19 to 7.03)		.46		1.79 (–2.41 to 5.99)		.40		1.00 (–3.08 to 5.08)			.63	
		0.4-0.8	7.53 (2.58 to 12.48)		.003		6.97 (2.70 to 11.24)		.001		5.16 (.99 to 9.34)			.02	
		0.9-4.7	13.05 (8.00 to 18.10)		<.001		10.45 (6.00 to 14.91)		<.001		8.41 (4.05 to 12.76)			<.001	
	**TOF**					.01^c^				.002^c^					.01^c^
		0.07-0.1	Ref				Ref				Ref				
		0.2-0.3	1.29 (–7.48 to 10.05)		.77		3.27 (–3.46 to 10.01)		.34		2.64 (–3.99 to 9.27)			.44	
		0.4-0.8	6.06 (–2.50 to 14.63)		.17		12.24 (5.31 to 19.18)		<.001		9.92 (3.07 to 16.78)			.005	
		0.9-4.7	10.09 (1.39 to 18.79)		.02		12.71 (5.53 to 19.90)		<.001		10.34 (3.23 to 17.45)			.004	
	**BMC^i^**					<.001^c^				<.001^c^					<.001^c^
		0.07-0.1	Ref				Ref				Ref				
		0.2-0.3	–.04 (–.66 to .58)		.90		–.10 (–.72 to .51)		.74		–.01 (–.62 to .60)			.97	
		0.4-0.8	–1.21 (–1.82 to –.60)		<.001		–1.21 (–1.85 to –.58)		<.001		–1.00 (–1.63 to –.38)			.002	
		0.9-4.7	–1.75 (–2.37 to –1.13)		<.001		–1.70 (–2.36 to –1.05)		<.001		–1.46 (–2.11 to –.81)			<.001	

^a^Model I was adjusted for gender, age, race, energy, creatinine, and urine.

^b^Model II was adjusted for gender, age, race, ratio of family income to poverty, education level, marital status, CAD score, smoking, alcohol intake per day, physical activity, energy, and creatinine, urine.

^c^*P* value for trend.

^d^Ref: reference.

^e^TLM: total lean mass.

^f^ALM: appendicular lean mass.

^g^TRF: trunk fat.

^h^TOF: total fat.

^i^BMC: bone mineral content.

The results of the interaction and stratified analyses are shown in Table 3. The associations between BPA, BPF, and body compositions were not significantly modified by gender. However, the association between BPS and body composition parameters (TLM, TRF, and TOF) was stronger among female participants (for interaction: *P*=.002, *P*=.007, and *P*=.003, respectively). A dose-response relationship was found between BPS and body composition parameters (TLM, TRF, and TOF; for trend *P*<.001, *P*<.001, and *P*=.001, respectively). In addition, the association of BPA and BPS with BMI was significantly modified by age (for interaction, *P*=.02 and *P*=.04, respectively). BPA was inversely associated with BMI in patients aged ≤20 years (for trend, *P*<.001). Compared with quartile 1, the BMI level decreased by 1.11 in quartile 4 (95% CI –1.61 to –.62; *P*<.001), whereas there was a dose-response association between BPA and BMI in people aged 20 to 50 years (*P*=.01, *P*<.001, and *P*=.003, respectively). For BPS, the association between BPS and BMI was stronger in people aged 20 to 50 years, and a dose-response association was found (for trend, *P*=.02). Furthermore, alcohol intake was a potential confounder in the association between BPF and ALM (for interaction, *P*=.001). Finally, the observed associations between bisphenols (BPA, BPS, and BPF) and body composition parameters were not significantly modified by race, smoking status, and physical activity (data not shown; Multimedia Appendix 2).

**Table 3:** Interaction and stratified analyses among American adults from the National Health and Nutrition Examination Survey 2003-2016.

						Quartile 1			Quartile 2							Quartile 3							Quartile 4							*P* value (trend)				*P* value (interaction)	
									β (95% CI)			*P* value			β (95% CI)				*P* value			β (95% CI)				*P* value									
**Urinary bisphenol A^a^**																																			
	**BMI (kg/m^2^; per age in years)**																																		.02
		Age ≤20			0			–.69 (–1.13 to –.25)			.002			–.87 (–1.33 to –.41)				<.001			–1.11 (–1.61 to –.62)				<.001			<.001							
		Age 20-50			0			.64 (.13 to 1.16)			.01			1.19 (.64 to 1.74)				<.001			.90 (.31 to 1.50)				.003			.04							
		Age ≥50			0			.12 (–.34 to .58)			.61			.23 (–.25 to .71)				.35			–.28 (–.79 to .24)				.30			.13							
**Urinary bisphenol S^b^**																																			
	**BMI (kg/m^2^; per age in years)**																																		.04
		Age ≤20			0			–.53 (–1.31 to .26)			.19			–.73 (–1.58 to .11)				.09			–.36 (–1.18 to .46)				.39			.89							
		Age 20-50			0			.26 (–.77 to 1.29)			.62			1.45 (.36 to 2.55)				.009			1.34 (.21 to 2.48)				.02			.02							
		Age ≥50			0			.37 (–.55 to 1.29)			.43			1.11 (.18 to 2.05)				.02			.69 (–.27 to 1.66)				.16			.39							
	**TLM^c^**																																		.002
		Male			0			–4.91 (–15.02 to 5.20)			.34			–5.49 (–15.61 to 4.63)				.29			–9.11 (–19.68 to 1.45)				.09			.15							
		Female			0			3.47 (–5.16 to 12.10)			.43			–3.19 (–12.06 to 5.68)				.48			–11.26 (–20.43 to –2.08)				.02			<.001							
	**TRF^d^**																																		.007
		Male			0			2.16 (–3.72 to 8.04)			.47			3.16 (–2.68 to 9.00)				.29			4.87 (–1.24 to 10.99)				.12			.16							
		Female			0			–2.59 (–8.40 to 3.22)			.38			1.67 (–4.24 to 7.59)				.58			9.93 (3.79 to 16.08)				.002			<.001							
	**TOF^e^**																																		.003
		Male			0			5.11 (–5.61 to 15.83)			.35			5.45 (–5.31 to 16.21)				.32			9.42 (–1.81 to 20.65)				.10			.16							
		Female			0			–4.78 (–13.98 to 4.42)			.31			3.98 (–5.46 to 13.42)				.41			11.19 (1.40 to 20.98)				.03			.001							
**Urinary bisphenol F^f^**																																			
	**ALM^g^**																																		.01
		**Alcohol intake**																																	
			None	0			–1.21 (–5.55 to 3.13)			.58			–.96 (–5.17 to 3.25)				.65			N/A^h^				N/A			.65								
			Moderate	0			–1.25 (–23.21 to 20.71)			.91			1.49 (–20.47 to 23.45)				.89			N/A				N/A			.69								
			Heavy	0			–9.23 (–24.19 to 5.73)			.23			–15.31 (–30.72 to .09)				.05			N/A				N/A			.28								

^a^Quartile ranges of urinary bisphenol A: quartile 1=0.14 to 0.7; quartile 2=0.8 to 1.5; quartile 3=1.6 to 3.1; quartile 4=3.2 to 11.

^b^Quartile ranges of urinary bisphenol S: quartile 1=0.07 to 0.1; quartile 2=0.2 to 0.3; quartile 3=0.4 to 0.8; quartile 4=0.9 to 4.7.

^c^TLM: total lean mass.

^d^TRF: trunk fat.

^e^TOF: total fat.

^f^Quartile ranges of urinary bisphenol F: quartile 1=0.3 to 0.6; quartile 2=0.7 to 7.5; quartile 3>7.5.

^g^ALM: appendicular lean mass.

^h^N/A: not applicable.

## Discussion

### Principal Findings

In this study, we discovered that there was no significant association between BPA and BMI, whereas BPA was negatively associated with TLM and ALM and had a positive dose-response with TOF and TRF. BPS was positively associated with BMI, TRF, and TOF and negatively associated with TLM, and there was no discernible relationship between BPS and ALM. However, no significant association was observed between BPF and body composition. Although previous studies have revealed that BPA is positively associated with human BMI [33-35], and urinary BPA content and phthalate metabolites are positively associated with body fat content in older adults and premenopausal women [1], it is unclear whether BPA and its derivatives (BPS and BPF) have similar effects on the specific body composition of the general population.

Eating habits may have influenced the study results. Wang et al [36] conducted a survey that included 360 primary and junior high school students in Shanghai as participants and found a positive association between BMI and BPA [36]. Regarding the possible effects of BPA exposure on adult BMI, the study by Carwile and Michels [33] that included 2747 adults participating in the 2003 to 2006 NHANES found that despite the nonlinear association between BPA and BMI, the BMI of the participants in the second, third, and fourth BPA quartiles were higher than those in the primary BPA quartile. Other studies investigated the relationship between BPA and BMI in young Indian women (age: mean 21.2, SD 2.37 years) and revealed that there was a significant association between BPA and BMI (*P*=.03) [37]. None of the 3 studies included diet as a possible covariate. In contrast, Braun et al [38] found no significant association between urinary BPA levels and BMI of prenatal women and 2-to 5-year-old children in the Cincinnati region after adjusting for diet, exercise, and di(2-ethylhexyl) phthalate. Thus, the differences among these findings may lie in whether diet was selected as a possible covariate and was well adjusted. Diet is an important source of exposure to BPA [39-41], whereas obesity is associated with certain dietary patterns, such as the Mediterranean diet [42] or ketogenic diet [43]. Previous studies indicated that consuming a substantial amount of plant-based foods in the Mediterranean diet may potentially reduce the severity of nonalcoholic fatty liver disease–related outcomes [44] and relate to favorable measures of adiposity [45]. Furthermore, a highly restrictive ketogenic diet is considered an effective and safe therapeutic intervention for patients who are obese [46,47], favoring their body composition [48]. In our study, total calorie intake, TOF intake, and alcohol intake were included as possible dietary covariates; the participants in the 2003 to 2004 and 2005 to 2006 NHANES were selected as participants of this study, and their body composition and urinary BPA level were well measured. No significant association was found between BMI and BPA in the fully adjusted model. This was consistent with the trend described by Braun et al [38].

There was a positive association between BPS and BMI, which is consistent with the findings of Jala et al [37]. In the study of the effect of BPS on male sperm quality, Ghayda et al [49] also found that men with high BMI have correspondingly higher urine BPS levels. For lean mass, both BPA and BPS showed a negative dose-response relationship with TLM, and there was a negative association between BPA and ALM in the fully adjusted model. Corbasson et al [1] found a negative association between BPA and TLM in the male group after adjusting for the covariates of waist circumference and BMI. However, this association was not observed in the female group. For body fat, urinary BPA and BPS levels in the NHANES participants were positively associated with TRF and TOF. Inconsistent with our findings, there was no significant relationship between BPA and fat mass in the study by Corbasson et al [1]. However, because of the use of NHANES as a data source for us and this study, our study incorporated an extended range of data periods, which may engender more inferences. Zhang et al [50] found that abnormal liver metabolism in mice, glycolysis, trichloroacetic acid metabolic pathway effects, and decreased succinic acid levels were related to visceral fat synthesis, indicating that BPS may also lead to an increase in TRF and TOF [51].

BPF was not significantly associated with TRF or TOF, possibly because current environmental BPF exposure is insufficient as a decisive factor for obesity. For example, Liu et al [52] used the data of 1521 participants in the NHANES 2013 to 2014 to evaluate the relationship between BPF and obesity. They also found that BPF and obesity were not significantly associated with obesity after adjusting for factors such as demographic, socioeconomic, lifestyle, and urinary creatinine concentration; however, a significant association was observed in the nonadjusted model. However, this might be an adjustment for sensitive populations, such as low-income individuals who are prone to overexposure. In their study, the exposure of BPF before and after adjustment was 0.4 versus 0.3 ng/mL, respectively, which was much less than the BPA exposure of 1.5 versus 1.1 ng/mL [52], and studies have shown that BPF can interfere with the IRS-1/PI3K/AKT signal transduction pathway and subsequently affect the insulin-dependent glucose metabolism pathway of adipocytes (3T3-L1 adipocytes), resulting in obesity [53].

According to the stratified analyses, the association between bisphenols and body composition was influenced by gender, age, and the potential impact of alcohol. Previous research has reported gender differences in the effects of BPA on obesity [54], and our study has also demonstrated such differences. On the basis of previous studies, this may be attributed to the differences in BPA metabolism and estrogen receptor expression, as well as gender-related BPA exposure [55,56]. Gender-related hormonal variations can lead to different responses to bisphenol exposure [57,58]. The Environmental Obesogen Hypothesis suggests that prenatal or early life exposure to estrogen-like environmental pollutants may increase the risk of fat accumulation and obesity [59]. For instance, BPA may have the greatest impact during early developmental stages, potentially due to epigenetic modifications related to stem cells [60]. In addition, alcohol consumption is associated with obesity, as it can affect liver metabolism and function [61]. The interaction between alcohol consumption and bisphenol compounds requires further research and validation.

Estrogen receptors play a crucial role in regulating lipid accumulation and improving insulin sensitivity and are therefore regarded as therapeutic targets for preventing obesity and metabolic disorders [57]. In vitro studies have previously observed that BPS and BPF exhibit similar toxicity and estrogenic effects as BPA, exerting an influence on cellular metabolism through pathways such as growth inhibition, oxidative stress, and gene expression [62]. Another study demonstrated the estrogenic agonistic effects of BPA, BPS, and BPF, individually, and the synergistic effect of these 3 bisphenols when combined, exhibiting a more potent action [63]. By contrast, obesity and metabolic disorders have been associated with oxidative stress [64]. Insulin resistance, a key factor in the pathogenesis of obesity and diabetes, is intricately linked to oxidative stress [65]. BPA and its derivatives, BPS and BPF, can disrupt the endogenous antioxidant system, leading to a severe imbalance in enzymes, such as superoxide dismutase and catalase, in liver cells exposed to BPS [66]. Furthermore, studies have indicated that adult exposure to BPF results in a significant imbalance in catalase activity [67]. Regarding the findings of this study, it is evident that both BPA and BPS have a significant impact on body composition, particularly on the content and distribution of adipose tissue. The potential mechanisms of BPS toxicity may involve interactions with estrogen receptors [68], DNA, and protein binding [69], as well as oxidative stress effects [70].

In this study, both BPA and BPS exhibited significant associations with body composition, displaying trends similar to those of BMI, TLM, TRF, and TOF. Furthermore, BPA was correlated with ALM, whereas BPS demonstrated an additional association with BMC. However, no significant correlation was observed between BPF and body composition. Studies suggest that BPS has a lesser obesogenic effect compared with BPA, with its ability to induce triglyceride accumulation being only 1% of that of BPA [71]. Nevertheless, for BPF exposure, some studies indicate an association with developing obesity, whereas others yield inconclusive results [72]; thus, further research is needed to elucidate its role.

In this cross-sectional study, although we were unable to establish a causal relationship between BPA and its derivatives, BPS and BPF, and body composition, we identified an association between them. The analysis conducted in this study not only provides a reference value for understanding the mechanisms of bisphenols but also holds potential value in guiding individuals to reduce adverse changes in body composition and mitigate metabolic-related reactions by proper exposure management or avoidance of bisphenols. Furthermore, it presents potential references for exploring the pathogenic mechanisms and preventive measures for obesity and other metabolic-related disorders. These findings underscore the importance of understanding the potential health risks associated with bisphenol exposure and emphasize the need for targeted interventions to mitigate the potential adverse effects on body composition.

Furthermore, to our knowledge, this is an innovative study to evaluate the association between specific body compositions, including ALM, TLM, TOF, TRF, and urinary BPA derivatives, such as BPS and BPF. Compared with previous studies, we included alcohol, calorie, and fat intake as possible covariates in the analysis to exclude the possible effects of diet on obesity based on the association between obesity and BPA as well as its derivatives. Thus, this study’s findings more accurately reflect the association between endocrine disrupters and body components. Another advantage of our study is that the American NHANES cohort has a large population base and ethnic diversity. Statistics was used for weighted analysis to represent a larger population. We also evaluated the association between BPS and TLM and found that BPS was negatively associated with TLM, which has not been previously reported. Lean body mass is significant for bone health and optimal aging [1]. The skeletal muscle is the largest and most malleable component of lean body mass (accounting for approximately 50% of lean body mass). Therefore, the change in lean body mass is mainly attributed to skeletal muscle mass. The maintenance of skeletal muscle is fundamental to locomotion, energy homeostasis, and overall quality of life [73]. This suggests that exposure to BPS may lead to a decline in exercise capacity, as the substitution of BPA also has safety issues. In fact, some studies have shown that BPS and BPA are positively associated with oxidative stress, which is associated with an increase in triglycerides and indirectly affects the physical fitness of healthy young adults (age: mean 23.5, SD 2 years). Jing et al [74] also found that the myotube diameter and distribution area of C2C12 myoblasts that were exposed to 10 to 4 M BPS decreased significantly compared with the control group.

### Limitations

Our study has several limitations. First, bisphenol exposure was measured using the urine of the NHANES participants. However, it is widely acknowledged that urinary BPA and its derivatives do not fully reflect bisphenol exposure. In fact, their levels change greatly over a short period, which may lead to uncertain relationships between BPA, BPS, BPF, and body composition [1]. Therefore, it is important to conduct repeated sampling measurements of the population. In addition, our covariates were limited and did not include other possible disrupters, such as exposure to other endocrine disrupters or drug intake, which can affect metabolism during the study period and the menopausal state of female individuals. Moreover, the data we used were collected from the urine of each participant only once, so it could not fully reflect the exposure to BPA and its derivatives. However, repeated sample collection may also have affected the enthusiasm of the participants, resulting in bias in the results. Nevertheless, urine collection is the most effective and widely adopted method for detecting BPA and BPS (bisphenol derivatives). The NHANES and other studies have generally used indirect methods to detect BPA levels. However, some studies have suggested that the bisphenol content in the human body may be disputable due to different detection methods. In 2020, a study proposed that direct methods measure BPA levels nearly 20 times higher than indirect methods, which may change our understanding of BPA levels in humans [75]. Nevertheless, the trend in the results obtained using mainstream detection methods is unquestionable. We found a negative dose-response between BPF, BPA, and TLM, which provides a basis for future research. However, the specific mechanism remains unclear and should be further explored in future studies. Additionally, the characteristics of human exposure to bisphenols are mixed. In this study, we analyzed the independent records of bisphenol compounds in the database, and we will analyze the effects of all bisphenol mixtures together by experimental methods for future research.

### Conclusions

Our study found that BPA, as a representative bisphenols, is related to changes in body composition. Although people have begun to pay more attention to BPA, the impact of its derivatives on humans cannot be ignored. As a derivative of BPA, BPS is related to the changes in various body components and has a stronger association with women and changes in age. BPF was not significantly associated with various body components in this study. We speculate that the degree of environmental exposure to BPF is not sufficient to cause a significant impact, but this conjecture needs to be elucidated through additional experiments.

## Appendix

Multimedia Appendix 1 All results of the associations between bisphenols and body composition among American adults that were obtained from the National Health and Nutrition Examination Survey 2003 to 2016.

Multimedia Appendix 2 All results of the interaction and stratified analyses between bisphenols and body composition among American adults from the National Health and Nutrition Examination Survey 2003 to 2016.

## Abbreviations

ALM: appendicular lean mass
BMC: bone mineral content
BPA: bisphenol A
BPF: bisphenol F
BPS: bisphenol S
CAD: coronary artery disease
DXA: dual-energy X-ray absorptiometry
NHANES: National Health and Nutrition Examination Survey
TLM: total lean mass
TOF: total fat
TRF: trunk fat

## Data Availability

The data used for this study were obtained from the National Health and Nutrition Examination Survey (NHANES) database, 2003 to 2016 [23], and only publicly available data were used in this study. The datasets generated or analyzed during this study are available from the corresponding author upon reasonable request.
