# Supplementary material for: Association Between Urinary Bisphenols and Body Composition Among American Adults: Cross-Sectional National Health and Nutrition Examination Survey Study
Source: JMIR Public Health Surveill. 2023 Sep 19;9:e49652. doi: 10.2196/49652 (PMC10548327; doi:10.2196/49652)
Supplement: Multimedia Appendix 1 [file publichealth_v9i1e49652_app1.doc]

| **Supplemental Table 1 All results of the associations between bisphenols and body composition among American adults from the National Health and Nutrition Examination Survey 2003-2016.** | | | |
| --- | --- | --- | --- |
|  | **Non-adjusted** | **Adjust Model I** | **Adjust Model II** |
| **Urinary Bisphenol A** | | | |
| **BMI** |  |  |  |
| 0.14 - 0.7 | ref | ref | ref |
| 0.8 - 1.5 | 0.62 (0.31, 0.92) <.001 | 0.31 (0.02, 0.59) 0.04 | 0.19 (-0.09, 0.46) 0.19 |
| 1.6 - 3.1 | 1.19 (0.89, 1.49) <.001 | 0.46 (0.16, 0.76) 0.002 | 0.35 (0.06, 0.64) 0.02 |
| 3.2 - 11 | 1.01 (0.70, 1.31) <.001 | -0.01 (-0.34, 0.32) 0.95 | -0.11 (-0.43, 0.20) 0.48 |
| *P* for trend | <.001 | 0.23 | 0.11 |
| **TLM** |  |  |  |
| 0.14 - 0.7 | ref | ref | ref |
| 0.8 - 1.5 | -1.65 (-6.58, 3.28) 0.51 | -8.96 (-12.66, -5.26) <.001 | -7.85 (-11.44, -4.25) <.001 |
| 1.6 - 3.1 | -2.15 (-7.05, 2.76) 0.39 | -13.17 (-17.06, -9.28) <.001 | -12.33 (-16.12, -8.54) <.001 |
| 3.2 - 11 | 3.00 (-1.98, 7.97) 0.24 | -11.62 (-15.79, -7.45) <.001 | -11.08 (-15.16, -7.01) <.001 |
| *P* for trend | 0.11 | <.001 | <.001 |
| **ALM** |  |  |  |
| 0.14 - 0.7 | ref | ref | ref |
| 0.8 - 1.5 | 1.47 (-1.63, 4.58) 0.35 | -4.07 (-6.53, -1.61) 0.001 | -3.31 (-5.73, -0.90) 0.007 |
| 1.6 - 3.1 | 2.93 (-0.17, 6.02) 0.0638 | -5.85 (-8.44, -3.25) <.001 | -5.46 (-8.01, -2.90) <.001 |
| 3.2 - 11 | 6.33 (3.19, 9.47) <.001 | -5.43 (-8.22, -2.65) <.001 | -4.80 (-7.55, -2.05) <.001 |
| *P* for trend | <.001 | 0.005 | 0.01 |
| **TRF** |  |  |  |
| 0.14 - 0.7 | ref | ref | ref |
| 0.8 - 1.5 | 1.63 (-1.37, 4.63) 0.29 | 4.53 (2.06, 7.00) <.001 | 3.55 (1.17, 5.93) 0.004 |
| 1.6 - 3.1 | 5.78 (2.79, 8.76) <.001 | 9.03 (6.44, 11.63) <.001 | 8.29 (5.78, 10.79) <.001 |
| 3.2 - 11 | 0.76 (-2.27, 3.79) 0.62 | 6.66 (3.88, 9.45) <.001 | 5.68 (2.98, 8.37) <.001 |
| *P* for trend | 0.85 | <.001 | 0.002 |
| **TOF** |  |  |  |
| 0.14 - 0.7 | ref | ref | ref |
| 0.8 - 1.5 | 2.13 (-2.99, 7.25) 0.42 | 9.66 (5.76, 13.57) <.001 | 8.58 (4.78, 12.39) <.001 |
| 1.6 - 3.1 | 3.73 (-1.37, 8.83) 0.15 | 15.07 (10.96, 19.18) <.001 | 14.19 (10.18, 18.20) <.001 |
| 3.2 - 11 | -2.04 (-7.21, 3.12) 0.44 | 13.08 (8.68, 17.48) <.001 | 12.51 (8.20, 16.82) <.001 |
| *P* for trend | 0.23 | <.001 | <.001 |
| **Urinary Bisphenol S** | | | |
| **BMI** |  |  |  |
| 0.07 - 0.1 | ref | ref | ref |
| 0.2 - 0.3 | 0.73 (0.13, 1.34) 0.02 | 0.27 (-0.30, 0.84) 0.35 | 0.26 (-0.29, 0.81) 0.35 |
| 0.4 - 0.8 | 2.02 (1.42, 2.62) <.001 | 1.04 (0.45, 1.62) <.001 | 0.91 (0.34, 1.48) 0.002 |
| 0.9 - 4.7 | 2.70 (2.10, 3.31) <.001 | 1.31 (0.70, 1.91) <.001 | 1.15 (0.55, 1.74) <.001 |
| *P* for trend | <.001 | <.001 | <.001 |
| **TLM** |  |  |  |
| 0.07 - 0.1 | ref | ref | ref |
| 0.2 - 0.3 | -2.53 (-10.95, 5.88) 0.56 | -4.46 (-10.82, 1.89) 0.17 | -3.81 (-10.05, 2.44) 0.23 |
| 0.4 - 0.8 | -6.42 (-14.64, 1.80) 0.13 | -12.79 (-19.32, -6.25) <.001 | -10.53 (-16.98, -4.08) 0.001 |
| 0.9 - 4.7 | -11.26 (-19.59, -2.92) 0.008 | -13.51 (-20.28, -6.74) <.001 | -11.14 (-17.83, -4.45) 0.001 |
| *P* for trend | 0.006 | <.001 | 0.005 |
| **TRF** |  |  |  |
| 0.07 - 0.1 | ref | ref | ref |
| 0.2 - 0.3 | 1.92 (-3.19, 7.03) 0.46 | 1.79 (-2.41, 5.99) 0.40 | 1.00 (-3.08, 5.08) 0.63 |
| 0.4 - 0.8 | 7.53 (2.58, 12.48) 0.003 | 6.97 (2.70, 11.24) 0.001 | 5.16 (0.99, 9.34) 0.02 |
| 0.9 - 4.7 | 13.05 (8.00, 18.10) <.001 | 10.45 (6.00, 14.91) <.001 | 8.41 (4.05, 12.76) 0.0002 |
| *P* for trend | <.001 | <.001 | <.001 |
| **TOF** |  |  |  |
| 0.07 - 0.1 | ref | ref | ref |
| 0.2 - 0.3 | 1.29 (-7.48, 10.05) 0.77 | 3.27 (-3.46, 10.01) 0.34 | 2.64 (-3.99, 9.27) 0.44 |
| 0.4 - 0.8 | 6.06 (-2.50, 14.63) 0.17 | 12.24 (5.31, 19.18) <.001 | 9.92 (3.07, 16.78) 0.005 |
| 0.9 - 4.7 | 10.09 (1.39, 18.79) 0.02 | 12.71 (5.53, 19.90) <.001 | 10.34 (3.23, 17.45) 0.004 |
| *P* for trend | 0.01 | 0.002 | 0.01 |
| **BMC** |  |  |  |
| 0.07 - 0.1 | ref | ref | ref |
| 0.2 - 0.3 | -0.04 (-0.66, 0.58) 0.90 | -0.10 (-0.72, 0.51) 0.74 | -0.01 (-0.62, 0.60) 0.97 |
| 0.4 - 0.8 | -1.21 (-1.82, -0.60) <.001 | -1.21 (-1.85, -0.58) 0.0002 | -1.00 (-1.63, -0.38) 0.002 |
| 0.9 - 4.7 | -1.75 (-2.37, -1.13) <.001 | -1.70 (-2.36, -1.05) <.001 | -1.46 (-2.11, -0.81) <.001 |
| *P* for trend | <.001 | <.001 | <.001 |
| β (95% CI) P-value was shown in the table. Adjust I model adjust for: Gender; Age; Race; Energy; Creatinine, urine. Adjust II model adjust for: Gender; Age; Race; Ratio of family income to poverty; Education level; Marital; CAD score; Smoking; Alcohol intake per day; Physical activity; Energy; Creatinine, urine. BMI = Body Mass Index, TLM = total lean mass, ALM = appendicular lean mass, TRF = trunk fat, BMC = bone mineral content, TOF = total fat. | | | |
