# Supplementary material for: Association Between Urinary Bisphenols and Body Composition Among American Adults: Cross-Sectional National Health and Nutrition Examination Survey Study
Source: JMIR Public Health Surveill. 2023 Sep 19;9:e49652. doi: 10.2196/49652 (PMC10548327; doi:10.2196/49652)
Supplement: Multimedia Appendix 2 [file publichealth_v9i1e49652_app2.doc]

| **Supplemental Table 2 All results of interaction and stratified analyses between bisphenols and body composition among American adults from the National Health and Nutrition Examination Survey 2003-2016.** | | | | | | |
| --- | --- | --- | --- | --- | --- | --- |
|  | **Quartile 1** | **Quartile 2** | **Quartile 3** | **Quartile 4** | **P for trend** | **P-interaction** |
| **Urinary Bisphenol A** | | | | | | |
| **BMI** |  |  |  |  |  |  |
| male | 0 | -0.17 (-0.55, 0.22) 0.40 | -0.23 (-0.63, 0.16) 0.25 | -0.62 (-1.04, -0.20) 0.004 | 0.003 | 0.76 |
| female | 0 | 0.04 (-0.38, 0.47) 0.85 | 0.25 (-0.20, 0.69) 0.28 | -0.30 (-0.80, 0.19) 0.2299 | 0.09 |
| Age <=20 | 0 | -0.69 (-1.13, -0.25) 0.002 | -0.87 (-1.33, -0.41) <.001 | -1.11 (-1.61, -0.62) <.001 | <.001 | 0.02 |
| Age 20-50 | 0 | 0.64 (0.13, 1.16) 0.01 | 1.19 (0.64, 1.74) <.001 | 0.90 (0.31, 1.50) 0.003 | 0.04 |
| Age >=50 | 0 | 0.12 (-0.34, 0.58) 0.61 | 0.23 (-0.25, 0.71) 0.35 | -0.28 (-0.79, 0.24) 0.30 | 0.13 |
| alcohol intake (none) | 0 | 0.10 (-0.21, 0.42) 0.52 | 0.05 (-0.28, 0.38) 0.77 | -0.39 (-0.74, -0.03) 0.03 | 0.004 | 0.12 |
| alcohol intake (moderate) | 0 | 0.23 (-0.86, 1.32) 0.68 | -0.13 (-1.28, 1.02) 0.82 | 0.45 (-0.80, 1.70) 0.49 | 0.5 |
| alcohol intake (heavy) | 0 | -0.00 (-0.78, 0.78) 0.997 | 0.13 (-0.74, 1.00) 0.76 | -0.21 (-1.13, 0.72) 0.66 | 0.56 |
| **TLM** |  |  |  |  |  |  |
| male | 0 | -8.50 (-14.13, -2.87) 0.003 | -8.86 (-14.57, -3.15) 0.002 | -11.83 (-17.80, -5.86) <.001 | 0.002 | 0.7 |
| female | 0 | -6.10 (-11.45, -0.76) 0.03 | -5.23 (-10.85, 0.38) 0.07 | -5.40 (-11.52, 0.72) 0.08 | 0.41 |
| Age <=20 | 0 | -5.29 (-12.14, 1.56) 0.13 | -4.57 (-11.74, 2.60) 0.21 | -8.50 (-16.12, -0.87) 0.03 | 0.06 | 0.14 |
| Age 20-50 | 0 | -5.65 (-11.17, -0.13) 0.0449 | -9.12 (-15.10, -3.14) 0.0028 | -10.03 (-16.43, -3.62) 0.0022 | 0.01 |
| Age >=50 | 0 | -5.45 (-12.74, 1.84) 0.14 | -4.61 (-12.05, 2.83) 0.22 | -9.17 (-17.10, -1.23) 0.02 | 0.04 |
| alcohol intake (none) | 0 | -7.58 (-11.95, -3.21) <.001 | -7.17 (-11.70, -2.64) 0.002 | -8.99 (-13.82, -4.16) <.001 | 0.01 | 0.16 |
| alcohol intake (moderate) | 0 | 1.21 (-13.70, 16.12) 0.87 | -1.04 (-16.74, 14.66) 0.90 | -1.55 (-18.55, 15.45) 0.86 | 0.77 |
| alcohol intake (heavy) | 0 | -8.37 (-18.65, 1.90) 0.11 | -8.90 (-20.45, 2.64) 0.13 | -12.43 (-24.23, -0.63) 0.04 | 0.1 |
| **ALM** |  |  |  |  |  |  |
| male | 0 | -4.72 (-8.73, -0.70) 0.02 | -4.45 (-8.53, -0.37) 0.03 | -5.78 (-10.06, -1.51) 0.008 | 0.048 | 0.8 |
| female | 0 | -1.60 (-4.45, 1.26) 0.27 | -1.73 (-4.73, 1.27) 0.26 | -0.85 (-4.12, 2.42) 0.61 | 0.96 |
| Age <=20 | 0 | -2.98 (-6.90, 0.93) 0.14 | -3.07 (-7.16, 1.03) 0.14 | -5.18 (-9.54, -0.82) 0.02 | 0.04 | 0.3 |
| Age 20-50 | 0 | 0.54 (-3.37, 4.46) 0.79 | 0.05 (-4.20, 4.31) 0.98 | -0.03 (-4.59, 4.53) 0.99 | 0.89 |
| Age >=50 | 0 | -2.28 (-7.24, 2.68) 0.37 | -4.71 (-9.77, 0.34) 0.07 | -4.54 (-9.95, 0.88) 0.10 | 0.15 |
| alcohol intake (none) | 0 | -2.87 (-5.54, -0.21) 0.03 | -3.40 (-6.17, -0.63) 0.02 | -4.15 (-7.11, -1.20) 0.006 | 0.03 | 0.91 |
| alcohol intake (moderate) | 0 | 2.30 (-8.70, 13.29) 0.68 | 0.12 (-11.56, 11.80) 0.98 | 2.08 (-10.56, 14.72) 0.75 | 0.85 |
| alcohol intake (heavy) | 0 | -4.31 (-12.37, 3.74) 0.29 | -2.26 (-11.33, 6.81) 0.63 | -0.06 (-9.34, 9.22) 0.99 | 0.56 |
| **TRF** |  |  |  |  |  |  |
| male | 0 | 3.77 (0.41, 7.12) 0.03 | 6.40 (2.99, 9.80) <.001 | 6.00 (2.44, 9.56) 0.001 | 0.009 | 0.51 |
| female | 0 | 2.19 (-1.46, 5.83) 0.24 | 3.76 (-0.06, 7.58) 0.05 | 1.78 (-2.40, 5.95) 0.40 | 0.82 |
| Age <=20 | 0 | 1.28 (-2.71, 5.28) 0.53 | 2.55 (-1.63, 6.73) 0.23 | 4.27 (-0.18, 8.72) 0.06 | 0.06 | 0.39 |
| Age 20-50 | 0 | 2.52 (-1.28, 6.31) 0.19 | 7.00 (2.88, 11.11) <.001 | 5.00 (0.58, 9.42) 0.03 | 0.07 |
| Age >=50 | 0 | 4.61 (-0.59, 9.81) 0.08 | 4.40 (-0.87, 9.67) 0.10 | 4.40 (-1.24, 10.03) 0.13 | 0.33 |
| alcohol intake (none) | 0 | 2.69 (-0.05, 5.44) 0.05 | 4.60 (1.76, 7.45) 0.002 | 3.58 (0.54, 6.61) 0.02 | 0.11 | 0.12 |
| alcohol intake (moderate) | 0 | -0.55 (-10.07, 8.97) 0.91 | -0.04 (-10.18, 10.10) 0.99 | 1.80 (-9.22, 12.81) 0.75 | 0.65 |
| alcohol intake (heavy) | 0 | 7.72 (0.68, 14.75) 0.03 | 8.17 (0.31, 16.03) 0.04 | 9.57 (1.50, 17.64) 0.02 | 0.11 |
| **TOF** |  |  |  |  |  |  |
| male | 0 | 8.88 (2.90, 14.87) 0.004 | 10.07 (4.01, 16.14) 0.001 | 12.88 (6.53, 19.22) <.001 | 0.001 | 0.78 |
| female | 0 | 6.76 (1.10, 12.41) 0.02 | 7.19 (1.25, 13.13) 0.02 | 7.45 (0.98, 13.92) 0.02 | 0.17 |
| Age <=20 | 0 | 6.17 (-1.12, 13.46) 0.10 | 6.47 (-1.15, 14.10) 0.10 | 11.07 (2.96, 19.18) 0.008 | 0.02 | 0.14 |
| Age 20-50 | 0 | 6.29 (0.41, 12.17) 0.04 | 10.90 (4.53, 17.27) <.001 | 11.46 (4.65, 18.28) 0.001 | 0.005 |
| Age >=50 | 0 | 6.09 (-1.50, 13.68) 0.12 | 4.91 (-2.82, 12.64) 0.21 | 9.81 (1.57, 18.04) 0.02 | 0.04 |
| alcohol intake (none) | 0 | 8.43 (3.79, 13.08) <.001 | 8.79 (3.98, 13.61) <.001 | 10.84 (5.71, 15.96) <.001 | 0.002 | 0.2 |
| alcohol intake (moderate) | 0 | -0.94 (-16.56, 14.68) 0.91 | 1.37 (-15.10, 17.84) 0.87 | 1.03 (-16.78, 18.85) 0.91 | 0.85 |
| alcohol intake (heavy) | 0 | 7.71 (-3.16, 18.57) 0.16 | 10.13 (-2.07, 22.33) 0.10 | 13.20 (0.75, 25.65) 0.04 | 0.08 |
| **BMC** |  |  |  |  |  |  |
| male | 0 | -0.52 (-1.03, -0.01) 0.04 | -0.42 (-0.93, 0.10) 0.12 | -0.16 (-0.70, 0.38) 0.56 | 0.7 | 0.34 |
| female | 0 | -0.38 (-0.91, 0.15) 0.16 | -0.55 (-1.10, 0.01) 0.05 | -0.08 (-0.69, 0.52) 0.78 | 0.55 |
| Age <=20 | 0 | -0.49 (-1.07, 0.09) 0.10 | -0.35 (-0.95, 0.26) 0.26 | -0.48 (-1.13, 0.16) 0.14 | 0.35 | 0.19 |
| Age 20-50 | 0 | -0.42 (-0.99, 0.16) 0.16 | -0.58 (-1.20, 0.05) 0.07 | -0.01 (-0.67, 0.66) 0.98 | 0.44 |
| Age >=50 | 0 | 0.31 (-0.46, 1.08) 0.43 | -0.20 (-0.99, 0.58) 0.61 | 0.13 (-0.71, 0.96) 0.77 | 0.93 |
| alcohol intake (none) | 0 | -0.51 (-0.92, -0.11) 0.01 | -0.51 (-0.93, -0.09) 0.02 | -0.19 (-0.64, 0.26) 0.40 | 0.71 | 0.78 |
| alcohol intake (moderate) | 0 | 0.55 (-1.02, 2.12) 0.49 | 0.66 (-1.00, 2.32) 0.44 | 0.92 (-0.87, 2.71) 0.32 | 0.39 |
| alcohol intake (heavy) | 0 | -0.17 (-1.27, 0.92) 0.76 | -0.74 (-1.97, 0.49) 0.24 | -0.13 (-1.38, 1.13) 0.84 | 0.93 |
| **Urinary Bisphenol S** | | | | | | |
| **BMI** |  |  |  |  |  |  |
| male | 0 | 0.31 (-0.44, 1.07) 0.41 | 0.48 (-0.28, 1.23) 0.21 | 0.58 (-0.21, 1.36) 0.15 | 0.25 | 0.08 |
| female | 0 | -0.25 (-1.06, 0.56) 0.55 | 0.17 (-0.66, 1.00) 0.68 | 0.99 (0.13, 1.86) 0.02 | 0.001 |
| Age <=20 | 0 | -0.53 (-1.31, 0.26) 0.19 | -0.73 (-1.58, 0.11) 0.09 | -0.36 (-1.18, 0.46) 0.39 | 0.89 | 0.04 |
| Age 20-50 | 0 | 0.26 (-0.77, 1.29) 0.62 | 1.45 (0.36, 2.55) 0.009 | 1.34 (0.21, 2.48) 0.02 | 0.02 |
| Age >=50 | 0 | 0.37 (-0.55, 1.29) 0.43 | 1.11 (0.18, 2.05) 0.02 | 0.69 (-0.27, 1.66) 0.16 | 0.39 |
| alcohol intake (none) | 0 | -0.06 (-0.66, 0.54) 0.85 | 0.29 (-0.31, 0.90) 0.34 | 0.83 (0.20, 1.47) 0.01 | 0.001 | 0.38 |
| alcohol intake (moderate) | 0 | 0.37 (-2.07, 2.81) 0.77 | 0.30 (-2.12, 2.73) 0.81 | -0.05 (-2.57, 2.47) 0.97 | 0.8 |
| alcohol intake (heavy) | 0 | 1.16 (-0.62, 2.94) 0.20 | 1.00 (-0.85, 2.85) 0.29 | 1.77 (-0.09, 3.64) 0.06 | 0.14 |
| **TLM** |  |  |  |  |  |  |
| male | 0 | -4.91 (-15.02, 5.20) 0.34 | -5.49 (-15.61, 4.63) 0.29 | -9.11 (-19.68, 1.45) 0.09 | 0.15 | 0.002 |
| female | 0 | 3.47 (-5.16, 12.10) 0.43 | -3.19 (-12.06, 5.68) 0.48 | -11.26 (-20.43, -2.08) 0.02 | <.001 |
| Age <=20 | 0 | 4.05 (-7.11, 15.22) 0.48 | -1.10 (-13.00, 10.79) 0.86 | -6.45 (-18.11, 5.21) 0.28 | 0.08 | 0.41 |
| Age 20-50 | 0 | -3.41 (-12.43, 5.61) 0.46 | -10.52 (-20.25, -0.78) 0.03 | -12.46 (-22.42, -2.51) 0.01 | 0.01 |
| Age >=50 | 0 | -7.83 (-22.97, 7.31) 0.31 | -10.68 (-26.07, 4.71) 0.17 | -11.66 (-27.56, 4.24) 0.15 | 0.33 |
| alcohol intake (none) | 0 | 1.57 (-5.83, 8.97) 0.68 | -2.66 (-10.10, 4.77) 0.48 | -10.40 (-18.16, -2.63) 0.009 | <.001 | 0.61 |
| alcohol intake (moderate) | 0 | 2.56 (-23.93, 29.06) 0.85 | -0.77 (-29.17, 27.62) 0.96 | 3.42 (-26.11, 32.96) 0.82 | 0.84 |
| alcohol intake (heavy) | 0 | -26.72 (-45.98, -7.47) 0.007 | -20.26 (-40.26, -0.26) 0.048 | -20.15 (-40.25, -0.04) 0.05 | 0.6 |
| **ALM** |  |  |  |  |  |  |
| male | 0 | -7.64 (-16.41, 1.14) 0.09 | -8.97 (-17.72, -0.21) 0.045 | -9.73 (-18.90, -0.55) 0.04 | 0.2 | 0.21 |
| female | 0 | -0.99 (-6.33, 4.34) 0.72 | -3.26 (-8.74, 2.23) 0.24 | -7.43 (-13.11, -1.75) 0.01 | 0.004 |
| Age <=20 | 0 | 0.14 (-7.12, 7.40) 0.97 | -4.75 (-12.47, 2.96) 0.23 | -4.99 (-12.57, 2.59) 0.20 | 0.13 | 0.92 |
| Age 20-50 | 0 | -6.97 (-14.59, 0.65) 0.07 | -9.28 (-17.48, -1.09) 0.03 | -9.65 (-18.09, -1.22) 0.03 | 0.14 |
| Age >=50 | 0 | -5.53 (-19.84, 8.78) 0.45 | -10.00 (-24.53, 4.54) 0.18 | -5.43 (-20.53, 9.66) 0.48 | 0.93 |
| alcohol intake (none) | 0 | -2.87 (-8.23, 2.49) 0.29 | -4.79 (-10.16, 0.59) 0.08 | -7.53 (-13.16, -1.90) 0.009 | 0.01 | 0.98 |
| alcohol intake (moderate) | 0 | 5.29 (-17.78, 28.36) 0.65 | -0.64 (-24.39, 23.11) 0.96 | 3.51 (-22.44, 29.46) 0.79 | 0.91 |
| alcohol intake (heavy) | 0 | -19.14 (-37.80, -0.49) 0.045 | -15.58 (-35.03, 3.86) 0.12 | -18.86 (-38.40, 0.69) 0.06 | 0.35 |
| **TRF** |  |  |  |  |  |  |
| male | 0 | 2.16 (-3.72, 8.04) 0.47 | 3.16 (-2.68, 9.00) 0.29 | 4.87 (-1.24, 10.99) 0.12 | 0.16 | 0.007 |
| female | 0 | -2.59 (-8.40, 3.22) 0.38 | 1.67 (-4.24, 7.59) 0.58 | 9.93 (3.79, 16.08) 0.002 | <.001 |
| Age <=20 | 0 | -2.83 (-9.25, 3.58) 0.39 | -1.90 (-8.69, 4.89) 0.58 | 3.25 (-3.44, 9.94) 0.34 | 0.08 | 0.91 |
| Age 20-50 | 0 | 1.54 (-4.53, 7.62) 0.62 | 7.88 (1.38, 14.38) 0.02 | 9.48 (2.80, 16.17) 0.006 | 0.003 |
| Age >=50 | 0 | 1.97 (-8.85, 12.78) 0.72 | 5.48 (-5.31, 16.28) 0.32 | 3.89 (-7.39, 15.17) 0.499 | 0.68 |
| alcohol intake (none) | 0 | -2.30 (-6.82, 2.22) 0.32 | 0.84 (-3.67, 5.35) 0.72 | 7.05 (2.33, 11.78) 0.004 | <.001 | 0.86 |
| alcohol intake (moderate) | 0 | -0.38 (-17.21, 16.46) 0.97 | 6.84 (-10.38, 24.06) 0.44 | 2.38 (-16.32, 21.09) 0.80 | 0.84 |
| alcohol intake (heavy) | 0 | 18.99 (5.80, 32.17) 0.005 | 13.32 (-0.28, 26.92) 0.06 | 17.55 (3.82, 31.29) 0.01 | 0.2 |
| **TOF** |  |  |  |  |  |  |
| male | 0 | 5.11 (-5.61, 15.83) 0.35 | 5.45 (-5.31, 16.21) 0.32 | 9.42 (-1.81, 20.65) 0.10 | 0.16 | 0.003 |
| female | 0 | -4.78 (-13.98, 4.42) 0.31 | 3.98 (-5.46, 13.42) 0.41 | 11.19 (1.40, 20.98) 0.03 | 0.001 |
| Age <=20 | 0 | -3.44 (-15.30, 8.41) 0.57 | 2.82 (-9.84, 15.47) 0.66 | 7.09 (-5.34, 19.52) 0.26 | 0.09 | 0.59 |
| Age 20-50 | 0 | 2.72 (-6.92, 12.37) 0.58 | 9.97 (-0.44, 20.37) 0.06 | 12.51 (1.86, 23.17) 0.02 | 0.02 |
| Age >=50 | 0 | 5.17 (-10.37, 20.71) 0.51 | 10.79 (-5.03, 26.62) 0.18 | 12.34 (-4.04, 28.72) 0.14 | 0.21 |
| alcohol intake (none) | 0 | -1.80 (-9.68, 6.07) 0.65 | 3.29 (-4.63, 11.21) 0.42 | 10.47 (2.18, 18.75) 0.01 | <.001 | 0.6 |
| alcohol intake (moderate) | 0 | -4.36 (-31.76, 23.04) 0.76 | -3.79 (-33.43, 25.86) 0.80 | -0.59 (-31.31, 30.12) 0.9698 | 0.88 |
| alcohol intake (heavy) | 0 | 25.39 (4.99, 45.78) 0.02 | 18.99 (-2.16, 40.15) 0.08 | 20.93 (-0.36, 42.21) 0.05 | 0.48 |
| **BMC** |  |  |  |  |  |  |
| male | 0 | -0.30 (-1.18, 0.59) 0.51 | -0.62 (-1.51, 0.27) 0.17 | -1.32 (-2.25, -0.39) 0.006 | 0.003 | 0.06 |
| female | 0 | 0.20 (-0.68, 1.08) 0.65 | -1.02 (-1.92, -0.12) 0.03 | -1.68 (-2.62, -0.75) <.001 | <.001 |
| Age <=20 | 0 | -0.17 (-1.09, 0.74) 0.71 | -0.86 (-1.84, 0.11) 0.08 | -1.51 (-2.47, -0.55) 0.002 | <.001 | 0.1476 |
| Age 20-50 | 0 | -0.06 (-1.02, 0.90) 0.90 | -1.03 (-2.06, 0.00) 0.05 | -1.54 (-2.59, -0.48) 0.004 | <.001 |
| Age >=50 | 0 | -0.11 (-1.65, 1.42) 0.89 | -1.18 (-2.74, 0.38) 0.14 | -1.27 (-2.88, 0.34) 0.12 | 0.11 |
| alcohol intake (none) | 0 | -0.01 (-0.69, 0.67) 0.97 | -0.87 (-1.56, -0.19) 0.01 | -1.72 (-2.43, -1.00) <.001 | <.001 | 0.83 |
| alcohol intake (moderate) | 0 | 1.73 (-1.05, 4.52) 0.22 | 0.77 (-2.24, 3.78) 0.62 | 0.14 (-2.98, 3.26) 0.93 | 0.58 |
| alcohol intake (heavy) | 0 | -1.38 (-3.39, 0.63) 0.18 | -1.04 (-3.13, 1.04) 0.33 | -1.36 (-3.45, 0.73) 0.20 | 0.5 |
| **Urinary Bisphenol F** | | | | | |  |
| **BMI** |  |  |  |  |  |  |
| male | 0 | 0.14 (-0.45, 0.73) 0.65 | -0.25 (-0.86, 0.36) 0.41 | / | 0.8 | 0.82 |
| female | 0 | 0.17 (-0.51, 0.85) 0.62 | 0.28 (-0.40, 0.95) 0.42 | / | 0.66 |
| Age <=20 | 0 | 0.24 (-0.39, 0.87) 0.46 | / | / | / | 0.33 |
| Age 20-50 | 0 | -0.40 (-1.19, 0.40) 0.33 | -0.23 (-1.10, 0.63) 0.60 | / | 0.18 |
| Age >=50 | 0 | 0.48 (-0.28, 1.24) 0.22 | 0.32 (-0.44, 1.08) 0.41 | / | 0.31 |
| alcohol intake (none) | 0 | 0.14 (-0.36, 0.64) 0.59 | 0.05 (-0.44, 0.54) 0.84 | / | 0.7 | 0.68 |
| alcohol intake (moderate) | 0 | 0.29 (-1.69, 2.27) 0.77 | 0.09 (-1.98, 2.16) 0.93 | / | 0.65 |
| alcohol intake (heavy) | 0 | 0.61 (-0.81, 2.03) 0.40 | -0.32 (-1.77, 1.12) 0.66 | / | 0.41 |
| **TLM** |  |  |  |  |  |  |
| male | 0 | -4.69 (-12.76, 3.38) 0.25 | -4.56 (-12.88, 3.76) 0.28 | / | 0.33 | 0.62 |
| female | 0 | -5.72 (-12.82, 1.38) 0.11 | -0.53 (-7.57, 6.51) 0.88 | / | 0.1 |
| Age <=20 | 0 | 0.44 (-8.28, 9.17) 0.92 | / |  | / | 0.007 |
| Age 20-50 | 0 | 0.75 (-6.24, 7.75) 0.83 | -1.13 (-8.91, 6.65) 0.78 | / | 0.63 |
| Age >=50 | 0 | -16.89 (-29.27, -4.50) 0.008 | -0.48 (-13.36, 12.40) 0.94 | / | 0.01 |
| alcohol intake (none) | 0 | -6.04 (-12.19, 0.10) 0.05 | -3.50 (-9.47, 2.47) 0.25 | / | 0.07 | 0.46 |
| alcohol intake (moderate) | 0 | -8.18 (-32.53, 16.17) 0.51 | -0.62 (-24.79, 23.55) 0.96 | / | 0.56 |
| alcohol intake (heavy) | 0 | -10.75 (-25.97, 4.48) 0.17 | 1.05 (-14.70, 16.79) 0.90 | / | 0.19 |
| **ALM** |  |  |  |  |  |  |
| male | 0 | -1.72 (-8.57, 5.14) 0.62 | -5.33 (-12.40, 1.73) 0.14 | / | 0.63 | 0.94 |
| female | 0 | -0.65 (-5.07, 3.76) 0.77 | -0.04 (-4.41, 4.33) 0.99 | / | 0.78 |
| Age <=20 | 0 | 0.31 (-5.29, 5.90) 0.91 | / |  | / | 0.02 |
| Age 20-50 | 0 | 2.01 (-3.86, 7.88) 0.50 | -2.12 (-8.58, 4.34) 0.52 | / | 0.42 |
| Age >=50 | 0 | -7.57 (-18.93, 3.80) 0.19 | -4.97 (-16.64, 6.69) 0.40 | / | 0.29 |
| alcohol intake (none) | 0 | -1.21 (-5.55, 3.13) 0.58 | -0.96 (-5.17, 3.25) 0.65 | / | 0.65 | 0.01 |
| alcohol intake (moderate) | 0 | -1.25 (-23.21, 20.71) 0.91 | 1.49 (-20.47, 23.45) 0.89 | / | 0.69 |
| alcohol intake (heavy) | 0 | -9.23 (-24.19, 5.73) 0.23 | -15.31 (-30.72, 0.09) 0.05 | / | 0.28 |
| **TRF** |  |  |  |  |  |  |
| male | 0 | 1.98 (-2.64, 6.60) 0.40 | 2.17 (-2.57, 6.92) 0.37 | / | 0.53 | 0.85 |
| female | 0 | 3.04 (-1.73, 7.81) 0.21 | 0.12 (-4.62, 4.86) 0.96 | / | 0.24 |
| Age <=20 | 0 | 0.91 (-4.11, 5.93) 0.72 | / |  | / | 0.41 |
| Age 20-50 | 0 | 0.69 (-4.00, 5.38) 0.77 | 0.86 (-4.27, 5.99) 0.74 | / | 0.999 |
| Age >=50 | 0 | 5.63 (-2.98, 14.24) 0.20 | 0.54 (-8.54, 9.63) 0.91 | / | 0.34 |
| alcohol intake (none) | 0 | 3.01 (-0.72, 6.73) 0.11 | 1.99 (-1.64, 5.62) 0.28 | / | 0.16 | 0.76 |
| alcohol intake (moderate) | 0 | 9.68 (-5.47, 24.84) 0.21 | 0.39 (-14.46, 15.24) 0.96 | / | 0.27 |
| alcohol intake (heavy) | 0 | 4.69 (-5.55, 14.92) 0.37 | -2.91 (-13.31, 7.48) 0.58 | / | 0.45 |
| **TOF** |  |  |  |  |  |  |
| male | 0 | 4.78 (-3.82, 13.38) 0.28 | 3.83 (-4.99, 12.66) 0.40 | / | 0.37 | 0.63 |
| female | 0 | 5.22 (-2.38, 12.83) 0.18 | -0.11 (-7.65, 7.43) 0.98 | / | 0.16 |
| Age <=20 | 0 | -0.53 (-9.82, 8.76) 0.91 | / |  | / | 0.01 |
| Age 20-50 | 0 | -1.63 (-9.13, 5.87) 0.67 | -0.45 (-8.77, 7.88) 0.92 | / | 0.498 |
| Age >=50 | 0 | 15.35 (2.42, 28.29) 0.02 | 0.80 (-12.61, 14.21) 0.91 | / | 0.03 |
| alcohol intake (none) | 0 | 5.84 (-0.73, 12.42) 0.08 | 3.07 (-3.30, 9.45) 0.34 | / | 0.11 | 0.6 |
| alcohol intake (moderate) | 0 | 11.89 (-14.00, 37.78) 0.37 | 2.40 (-22.77, 27.58) 0.85 | / | 0.47 |
| alcohol intake (heavy) | 0 | 12.10 (-3.89, 28.10) 0.14 | -1.29 (-17.83, 15.26) 0.88 | / | 0.16 |
| **BMC** |  |  |  |  |  |  |
| male | 0 | -0.44 (-1.16, 0.28) 0.23 | -0.00 (-0.74, 0.74) 0.9998 | / | 0.37 | 0.96 |
| female | 0 | -0.10 (-0.82, 0.63) 0.79 | 0.07 (-0.65, 0.79) 0.84 | / | 0.74 |
| Age <=20 | 0 | -0.14 (-0.86, 0.58) 0.71 | / |  | / | 0.16 |
| Age 20-50 | 0 | 0.14 (-0.61, 0.89) 0.71 | 0.27 (-0.56, 1.10) 0.52 | / | 0.47 |
| Age >=50 | 0 | -0.43 (-1.71, 0.86) 0.52 | 0.35 (-0.98, 1.67) 0.61 | / | 0.67 |
| alcohol intake (none) | 0 | -0.33 (-0.90, 0.24) 0.26 | -0.22 (-0.77, 0.34) 0.45 | / | 0.4 | 0.57 |
| alcohol intake (moderate) | 0 | 0.22 (-2.42, 2.86) 0.87 | 1.07 (-1.50, 3.64) 0.42 | / | 0.95 |
| alcohol intake (heavy) | 0 | 0.10 (-1.48, 1.68) 0.90 | 0.83 (-0.80, 2.47) 0.32 | / | 0.98 |
| β (95% CI) P-value was shown in the table. Quartile ranges of Urinary Bisphenol A: Quartile 1=0.14 to 0.7; Quartile 2=0.8 to 1.5; Quartile 3=1.6 to 3.1; Quartile 4: 3.2 to 11. Quartile ranges of Urinary Bisphenol S: Quartile 1=0.07 to 0.1; Quartile 2=0.2 to 0.3; Quartile 3=0.4 to 0.8; Quartile 4: 0.9 to 4.7. Quartile ranges of Urinary Bisphenol F: Quartile 1=0.3 to 0.6; Quartile 2=0.7 to 7.5; Quartile 3 >7.5. BMI = Body Mass Index, TLM = total lean mass, ALM = apendicular lean mass, TRF = trunk fat, BMC = bone mineral content, TOF = total fat. | | | | | | |
